# Rhodanine to Oxorhodanine Switch Switches Switching Mechanism in a Monomethine Photoswitch

**DOI:** 10.1021/acs.jpclett.6c00690

**Published:** 2026-06-03

**Authors:** Dipanjan Banerjee, Pratip Chakraborty, Anam Fatima, Giovanni Bressan, Erico M. Braun, Isabelle Chambrier, Garth A. Jones, James N. Bull, Andrew N. Cammidge, Stephen R. Meech

**Affiliations:** † Chemistry Department, School of Chemistry, Pharmacy and Pharmacology, 6106 University of East Anglia, Norwich NR4 7TJ, U.K.; ‡ Instituto de Física, Universidade Federal do Rio Grande do Sul, Av. Bento Gonçalves, 9500 Porto Alegre, Brazil

## Abstract

Understanding the excited-state dynamics of molecular photoswitches is key for advancing their design and optimizing their applications. Here, we characterize the excited-state chemistry of a recently reported oxorhodanine photoswitch through ultrafast spectroscopy and multireference quantum chemical calculations. Both *Z* and *E* forms undergo excited-state isomerization reactions on a sub-picosecond time scale to form a hot ground-state and the product isomer. The reaction is shown to proceed entirely within the singlet manifold, in sharp contrast to the rhodanine photoswitches, which react through the triplet state. The difference is ascribed to the *n*π* state arising from the C=S bond in the rhodanines. The dominance of ultrafast relaxation in the singlet state is confirmed by multireference *ab initio* calculations which also show that the reaction coordinate involves torsion and pyramidialization. This reaction coordinate is consistent with the observed viscosity dependence. Calculations also indicate that the observed differences between ultrafast relaxation in the *Z* and *E* forms may arise from a shallow minimum on the excited-state of the latter.

## Graphical abstract

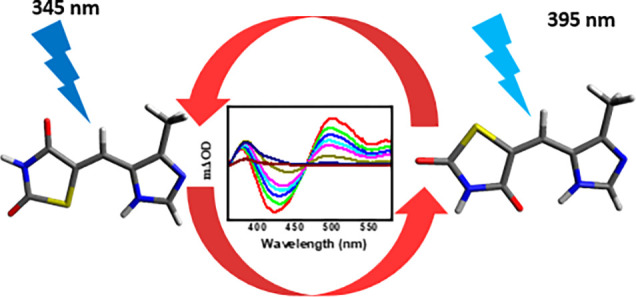

Reversible optical switching of a system between two states is critical in photobiology, photopharmacology and bioimaging and in numerous applications in photonics and organic materials.^1–3^ Consequently, there has been intense research into the synthesis, characterization and optimization of molecular photoswitches.^4–7^ Critical objectives include improving switching rates and yields, enhancing photostability, tuning the switching wavelength to lower energy and clearly separating forward and reverse switching wavelengths. Quite recently a new family of photoswitches, the rhodanines and oxorhodanines, was synthesized and characterized by Köttner et al.^8^ These monomethine photoswitches are robust, have UV–visible switching wavelengths and high quantum yields. It was demonstrated that in one example photoswitching induced apoptosis in HeLa cell.^8^

Previously, we investigated the switching mechanism and excited-state dynamics of a typical example of a rhodanine photoswitch (**II** in Figure 1).^9^ In that work, an unexpectedly long-lived (hundreds of picoseconds) nonemissive intermediate was detected and characterized through transient electronic and vibrational spectroscopy. Modeling those data through high level multireference quantum chemical calculations showed that an unusual triplet mediated photoswitching mechanism was operating.^9^ This result has implications for photoswitch photostability and possibly for the mechanism operating in light activated cell death. In this letter, we describe a detailed experimental and theoretical investigation of photodynamics in the equivalent oxorhodanine photoswitch (**I**, the structures of the two photoswitches are compared in Figure 1). The measurements show that both excited-state relaxation and ground-state recovery in **I** are ultrafast, mediated by motion along an excited singlet state isomerization coordinate leading to a conical intersection (CoIn) with the electronic ground-state. Quantum chemical calculations further show that the different photophysics of **I** and **II** are due to a low-lying *n*π* state localized on the C=S bond of **II**. The singlet mediated mechanism operating in **I** is similar to some other monomethine dyes^10–13^ and analogous to the well-characterized photoreaction of thioindigoid photoswitches, where the dynamics are slower reflecting the presence of a barrier in the photoisomerization coordinate.^14,15^

**Figure 1:**
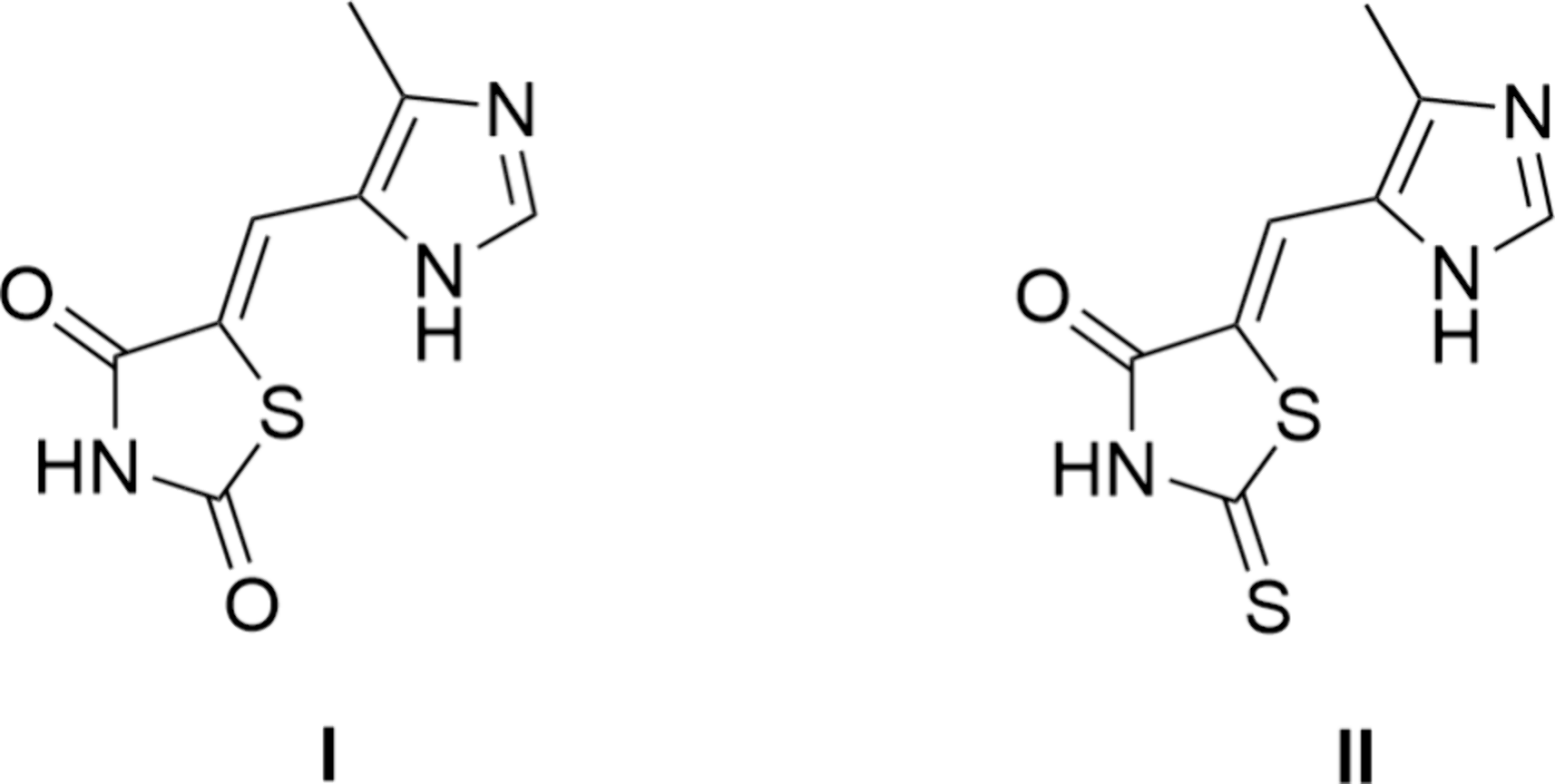
Oxorhodanine photoswitch (**I**) studied in this work, and rhodanine photoswitch (**II**) studied in ref 9.

The steady state data for **I** in three fluid solvents of different polarity and one polar viscous solvent are shown in Figure 2. The emission spectra normalized to the absorbance at the excitation wavelength are weak (estimated fluorescence quantum yield of 10^–4^) and quite broad. The absorption spectra in methanol (MeOH) and ethylene glycol (EG) are slightly red-shifted and broadened compared to acetonitrile, while in the least-polar solvent, THF, the absorption shows weak structure; however, overall, the polarity dependence is small. It is also evident that the relative emission intensity in the viscous EG solvent is significantly enhanced, compared to fluid solvents. Continuous irradiation at 340 nm leads to a permanent red shift in the absorption, assigned to formation of the *E* isomer through an excited-state isomerization reaction (see Figure S1 in the Supporting Information (SI)).

**Figure 2:**
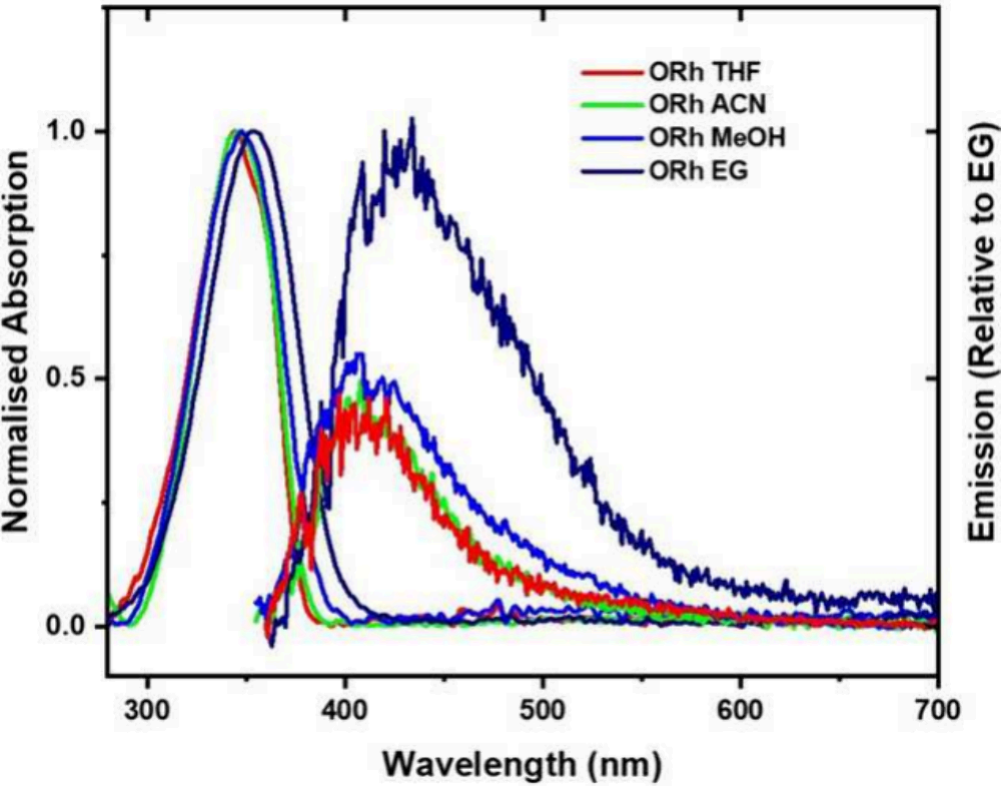
Steady-state absorption and emission spectra (λ_exc_ = 345 nm) of **I** in different solvents at room temperature (solvent Raman contributions were subtracted from the emission). Emission spectra are adjusted to correct for variations in sample absorbance and presented relative to ethylene glycol (EG) to indicate the relative fluorescence yield.

The transient absorption (TA) data for **I** in acetonitrile (ACN) between 360 and 1000 nm are shown in Figure 3a. Within 100 fs of excitation at 340 nm, there is an obvious transient absorption above 470 nm that is slightly structured (shoulder at 560 nm). In addition, there is a very weak broad absorption near 1000 nm. These two transients are assigned to S_1_ → S_*n*_ transitions. Below 470 nm stimulated emission (SE) dominates the signal, this assignment being based on the emission spectrum (also shown in Figure 3a). Below 400 nm, a new transient absorption is observed. On a time scale of a few picoseconds, the TA and SE decay monotonically toward the baseline, although after 2 ps, there is a broadening of the TA below 400 nm on the red side, which relaxes further to a long-lived (i.e., with a lifetime longer than our few-ns time range) product.

**Figure 3:**
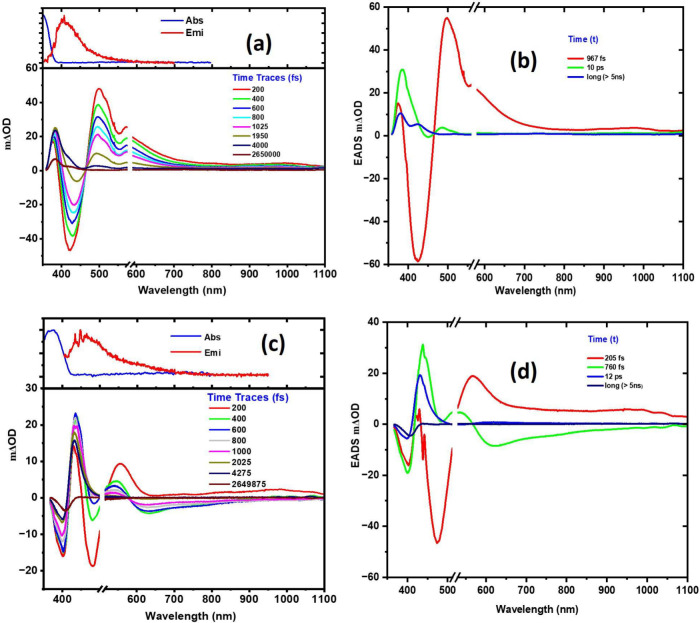
(a) Transient absorption spectra of the *Z* form of **I** in acetonitrile at different time delays after the 345 nm pump pulse, with steady-state absorption and emission spectra included above for comparison with ground-state bleach (GSB) and stimulated emission (SE). (b) Evolution-associated difference spectra (EADS) derived from sequential global fitting of the corresponding TA data for the *Z* form. (c) Transient absorption spectra of the *E* form of **I** in acetonitrile at different time delays after the pump pulse at 395 nm, with steady-state absorption and emission spectra included as for panel (a). (d) EADS for the *E* form. The axis break above 500 nm indicates where UV–vis and near-IR datasets join (see the SI). The *Y*-axis in all the figures is in mΔOD units.

A global analysis,^16^ in terms of a sequence of first-order steps, is informative, with the evolution-associated difference spectra (EADS) shown in Figure 3b. The dominant decay in acetonitrile occurs in 1 ps to leave a weaker transient absorption at ca. 400 nm which decays in 10 ps to reveal a long-lived transient absorption also at 400 nm; the latter is assigned to formation of the *E* isomer (see Figure S1). The initial subpicosecond step is assigned to the decay of the excited singlet state, consistent with the disappearance of the SE (Figure 3b). The intermediate 10 ps relaxation can be assigned to cooling of a hot ground-state formed by the ca. 1 ps internal conversion (IC). Similar transients occurring on the red edge of the ground-state absorption of related dyes have been reported.^17,18^ Recently, we presented a detailed study of IC and ground-state cooling in a similar monomethine dye, modeling the transient IR spectra.^19^ In that case, cooling also occurred on a few picosecond time scale, consistent with the current observation.

The TA data measured in the other solvents are shown in the SI (Figures S2–S5), along with EADS from the same sequential global analysis and the quality of the fit at some key wavelengths. The time constants recovered are shown in Table 1. For the *Z* isomer in MeOH solvents, the fast IC time are similar to those in ACN, and the second EADS can again be assigned to vibrational cooling,^19^ with a relaxation time that is only weakly solvent-dependent. However, in the polar but more viscous EG the decay of the SE is observed to be significantly slower (Figure S5 and S12a, consistent with the observed enhanced fluorescence yield in EG, Figure 2). As a consequence of the longer emissive state lifetime (Figure S12a) the SE contributes to both the τ_1_ and τ_2_ EADS. Consequently, τ_2_ in EG does not resolve the excited-state decay from the vibrational relaxation. Including an additional intermediate in the analysis did not improve the quality of fit significantly, so we conclude that, in EG, the second relaxation time (Table 1) reflects a mixture of excited-state decay and ground-state cooling occurring on the same few picoseconds time scale. Such an increase in excited-state lifetime with increasing viscosity has been observed in several^20–22^ (but by no means all^13,23^ ) molecules exhibiting excited-state photoisomerization. This viscosity dependence can be ascribed to a reaction coordinate which displaces a significant volume of solvent, such as an out-of-plane rotation of one of the aromatic groups about the bridging bonds.

**Table 1:** Solvent Parameters (Dielectric Constant, ϵ, and Viscosity, η) and the Time Constants Associated with Transitions between the EADS^a^

			Time Constants			
Isomer/Solvent	Dielectric Constant, ϵ	Viscosity, η (mPa s)	τ_1_ (ps)	τ_2_ (ps)	τ_3_ (ps)	τ_4_ (ns)
*Z*/ACN	35.9	0.344	1.0	10.0	–	>5
*Z*/MeOH	33.7	0.543	0.8	4.1	–	>5
*Z*/THF	7.58	0.456	1.6	4.3	–	>5
*Z*/EG	41.3	16.2	0.8	5.9	–	>5
*E*/ACN	35.9	0.344	0.2	0.8	12.0	>5

a “Long” times were beyond the optical delay range and fixed at 5 ns. The analysis of the *E* isomer of **I** required an additional relaxation component.

In the nonviscous less-polar solvent THF, the decay of the SE is lengthened compared to ACN (1.6 ps, compared to 1.0 ps) but much faster than in EG (Figure S12a), suggesting a weak polarity effect alongside the stronger viscosity effect. The medium polarity effect on the rate of excited state decay can arise from the charge separation that frequently occurs on twisting of a double bond.^24^ However, there are also qualitative differences in the early time SE of THF and ACN (and MeOH). Specifically, the SE of THF unexpectedly reveals an appearance time within 100 fs, right at the limit of our time resolution (Figure S12b). Global analysis with an additional component compared to ACN (Figure S11a,b) suggests this may be assigned to decay of an underlying transient absorption in ca. 50 fs. This component does not appear in the near IR TA and no such component was resolved in the other three more polar solvents. The assignment of this relaxation in THF is unclear. Quantum chemical calculations (see below) resolve two excited states separated by 0.45 eV for the *Z* isomer. The onset of absorption is at 380 nm while the excitation wavelength is 340 nm, so excitation in the region of the upper state is plausible. We speculate that there is an initial sub 100 fs internal conversion in THF which is accelerated in polar solvents. We note that the absorption spectrum in THF reveals slight structure (a shoulder at 345 nm) which is absent in the more polar solvents, consistent with faster IC in the polar solvents (or, equivalently, a stronger S_2_/S_1_ coupling).

Finally, a stationary state population of the *E* isomer in ACN was generated by CW irradiation at 340 nm. This *E* population was then selectively excited at 395 nm (see Figure S1 for spectra) and the emission spectrum and TA data were recorded. The emission spectrum is very broad, extending beyond 700 nm. The TA show the same essential components as the *Z* form with some notable differences. A ground-state bleach is now resolved near 400 nm which can be assigned to the absorption of the *E* form being red-shifted compared with the *Z* form by ∼30 nm (Figure S1), and thus appearing in the range of our continuum probe. The dominant fast SE is similarly red-shifted with respect to the *Z* isomer, and the NIR absorption is somewhat enhanced. Significantly a stimulated emission is observed to persist for longer times, between 600 nm and 800 nm. These data were analyzed with a sequential global kinetics model (Figure 3c,d). The quality of the fit at some key wavelengths has been shown (in ACN) in Figure S6. Although the behavior is similar to that of the *Z* isomer there are notable differences. The TA have the same initial features as the *Z* isomer, with a slightly enhanced TA at 1 μm. The short wavelength SE decays rapidly (ca. 200 fs) to leave a more red-shifted SE, which reflects the broader steady-state emission (Figure S6a). That SE then decays in 0.8 ps to leave a hot ground state, which cools in 12 ps. The final product in this case is a long-lived bleach, rather than a transient absorption, reflecting the blue shift in absorption during the *E* → *Z* reaction. The more complex spectra, including a red-shift in the SE with time and the nonsingle exponential excited-state decay for the *E* isomer suggest some additional relaxation differences in the excited-state dynamics between the *E* and *Z* isomers prior to accessing the hot ground state by IC through a CoIn.

To provide further insight into the excited-state dynamics of **I**, we performed quantum chemical calculations. Both *Z* (S_0_-min (Z)) and *E* (S_0_-min (E)) isomers of the photoswitch on the ground-state were optimized at the density functional theory (DFT)^25,26^ level, using the ωB97X-D^27^ functional and the 6–31G­(d,p)^28,29^ basis set. Frequency calculations were performed to ensure that the stationary points obtained are the minima. The S_1_/S_0_-minimum-energy CoIn (S_1_/S_0_-MECI) was optimized at the complete active space self-consisted field (CASSCF)^30^ level using cc-pVDZ^31^ basis set and an active space of 12 electrons in 11 orbitals (CAS­(12,11)). Vertical excitation energies and oscillator strengths were calculated at all the critical points and along the linearly interpolated pathways connecting them in internal coordinates (LIIC) at the extended multistate complete active space second-order perturbation (XMS-CASPT2)^32–34^ theory level using an active space of 14 electrons in 12 orbitals (CAS­(14,12)) and cc-pVDZ basis set. For a more detailed description of the computational methods and the active spaces employed, refer to the SI. The DFT calculations were carried out in Gaussian16,^35^ while the MECI optimization was performed in BAGEL 1.2.2 ^36,37^ and XMS-CASPT2 calculations along the LIIC were performed using OpenMolcas 25.10.^38^

The calculations are summarized in Figure 4. Comparing these results with those for rhodanine **II**,^9^ it is immediately clear that **I** is simpler due to the absence of a low-lying *n*π* state. In the rhodanine **II**, the *n*π* state was nearly degenerate with the ππ* state and crossed it in an ultrafast excited-state evolution, which resulted in population of the triplet state.^9^ From the earlier calculations, we can further conclude that the C=S bond in the rhodanine **II**, on which the low-lying *n*π* excitation was localized, is the source of the mechanistic difference. In contrast, the first excited state of **I** is an isolated singlet ππ* state with the lowest triplet ππ* state well-separated from it (Figure 4a). The LIIC pathway from the Franck–Condon (FC) excited state was calculated for both *Z* and *E* isomers (Figure 4a). While the initial *Z* ground state has a well-defined minimum energy at the planar configuration, the excited state is relatively flat. There is an energetically downhill pathway which initially involves twisting about the bridging bond. After ∼10 amu^1/2^·Å, pyramidalization at the oxorhodanine ring carbon also contributes to the LIIC pathway, which is accompanied by a steeper energy decline leading to a CoIn with the ground state. We thus assign the rapid excited state decay to this energetically downhill torsion/pyramidalization coordinate. The significant effect of viscosity on the decay time suggests that motion along the LIIC coordinate is opposed by solvent friction, which is consistent with the torsion about the bridging bond calculated (Figure 4a), which requires displacement of solvent molecules. As the reaction proceeds, the S_0_ → S_1_ transition dipole moment decreases (illustrated via decreasing oscillator strength of the S_1_ state from FC region toward the CoIn region in Figure 4c), as has been reported for related monomethine dyes undergoing excited-state torsion.^39^ The triplet state remains energetically well separated from the singlet excited state until very close to the CoIn, and the spin–orbit coupling remains small (<10 cm^–1^) compared to **II** (see Figure 4b and ref 9), although increasing closer to the CoIn as singlet and triplet approach. This excited-state mechanism is thus fully consistent with the ultrafast solvent-friction-dependent decay observed in the TA data (Figure 3).

**Figure 4:**
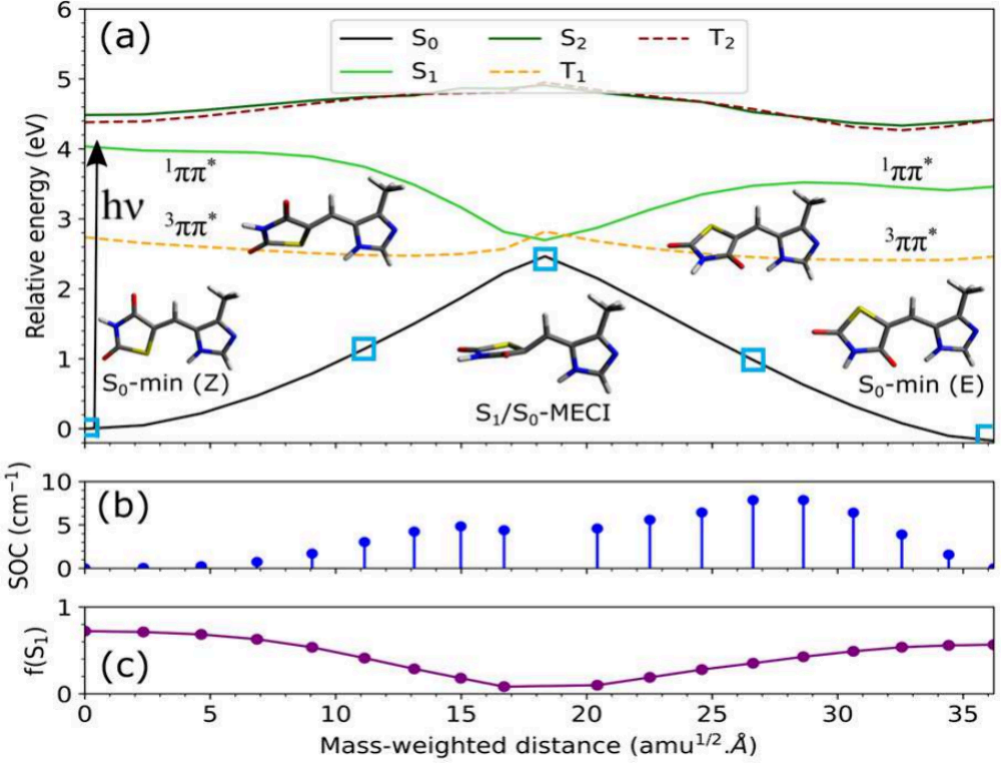
(a) Relative potential energies (with respect to S_0_-min (Z)) along the LIIC from S_0_-min (Z) to S_1_/S_0_-MECI, and from S_1_/S_0_-MECI to S_0_-min (E), calculated at the XMS-CASPT2/SA­(5/3)-CAS­(14,12)/cc-pVDZ level of theory along with the character of the states (*n*π* or ππ*) involved, where SA­(5/3) refers to averaging over five-singlet and three-triplet states. Three singlet states and two triplet states are illustrated using solid and dashed lines, respectively. S_1_/S_0_-MECI was optimized at SA2-CASSCF­(12,11)/cc-pVDZ level, which is why there is a small energy gap between S_1_ and S_0_ levels at S_1_/S_0_-MECI at the higher XMS-CASPT2 level. Three critical points and two geometries along the LIIC are shown associated with the blue squares on *x*-axis which is in mass-weighted distance (amu^1/2^·Å) relative to the S_0_-min (Z). (b) Spin–orbit coupling (SOC) (in cm^–1^) between S_1_ and T_1_ states along the same pathway calculated using the above-mentioned XMS-CASPT2 level. (c) Oscillator strength (*f*) of the S_1_ state along the same pathway calculated using the above-mentioned XMS-CASPT2 level. Due to state mixing at the S_1_/S_0_-MECI, we omitted the SOC and oscillator strengths at that point (∼18.3 amu^1/2^·Å).

There are some significant differences between the measured relaxation dynamics of the *Z* and *E* forms of **I** (Figure 3a,c). Most notably, in the *E* form, the SE appears to red shift over the first picosecond, which was not seen for the *Z* isomer. This correlates with the overall broader emission spectrum for *E* (Figure 3c, upper panel). It can be seen in Figure 4 that this difference may arise from subtle changes in the shape of the calculated LIIC surface, which are highlighted by the direct comparison in Figure S10. Specifically, the *E* form appears to have a very shallow minimum between 30 and 35 amu^1/2^·Å (Figure 4a). While not deep enough to trap the population for a significant length of time, the different LIIC surfaces may suggest a different evolution pathway for the *E* isomer, which relaxes on a subpicosecond time scale, as for *Z*, but remains in a region with a larger transition dipole moment for emission, allowing the red-shift in the SE to be resolved (Figure 3d). An alternative explanation for the origin of the red-shifted emission is a level inversion between S_2_ and S_1_ along the reaction coordinate. However, the calculation (Figure 4a) show an increasing rather than decreasing separation between these states.

In summary, the excited-state dynamics of the oxorhodanine photoswitch **I** have been measured by TA and simulated by quantum chemical calculations. The excited-state decay occurs within ca. 1 ps in fluid solvents and leads to population of a hot ground state, which relaxes in several picoseconds. The excited-state lifetime is significantly extended in viscous solvents. This behavior is quite different from, and simpler than, that of the corresponding rhodanine photoswitch, **II**. The calculations for **I** are consistent with an ultrafast relaxation along a LIIC pathway to a CoIn with the ground state. The LIIC coordinate involves torsion about the bridging bond and pyramidalization, consistent with the observed solvent viscosity effect. The calculations further explain the change in mechanism compared with the rhodanine photoswitch, which arises from the role of *n*π* state in the rhodanine localized on the C=S bond, which promotes intersystem crossing. Thus, although the rhodanine and oxorhodanine photoswitches are quite similar in their steady-state properties, they have underlying excited-state dynamics that occur on dramatically different time scales and allow them to access different electronic states. These effects will modify their interaction with any host medium.

## Experimental Details

The TA data were recorded on an instrument described elsewhere,^40,41^ with specific details of construction, wavelengths, and sample handling given in the SI. Quantum chemical calculations are detailed in the SI. The synthesis of **I** has been reported previously, but the details and analytical information are provided in the SI.

## Supplementary Material



## Notes

Additional experimental details and further details on quantum chemical calculations; additional data includes TA data on **I** for all solvents not shown in main text along with the corresponding EADS and examples of fitting, and active space of orbitals used for the quantum chemical calculations along with the Cartesian coordinates of important critical points required to construct the LIIC between them (PDF)
