# Supplementary material for: Rhodanine to Oxorhodanine Switch Switches Switching Mechanism in a Monomethine Photoswitch
Source: J Phys Chem Lett. 2026 Jun 3;17(24):6691–6. doi: 10.1021/acs.jpclett.6c00690 (PMC13288687; doi:10.1021/acs.jpclett.6c00690)
Supplement: Supplementary file 1 [file jz6c00690_si_001.pdf]

**Rhodanine to Oxorhodanine Switch Switches Switching Mechanism in a Monomethine Photoswitch**

*Dipanjan Banerjee*[†1], *Pratip Chakraborty*[†1], *Anam Fatima*[1], *Giovanni Bressan*[1], *Erico M. Braun*[2], *Isabelle Chambrier*[1], *Garth A. Jones*[1], *James N. Bull*[1], *Andrew N. Cammidge*[1], *and Stephen R. Meech**[1]

[1]Chemistry Department, School of Chemistry, Pharmacy and Pharmacology, University of East Anglia, Norwich NR4 7TJ, UK

[2]Instituto de Física, Universidade Federal do Rio Grande do Sul, Av. Bento Gonçalves, 9500, Porto Alegre, Brazil

†These authors contributed equally to the work

*Address for correspondence: s.meech@uea.ac.uk

# I. Contents

**1. Experimental section** ........ 4

**a) Steady-State Absorption and Emission** ........ 4

**b) UV-Visible-NIR fs transient absorption (TA)** ........ 4

**c) Fluorescence Quantum Yield.** ........ 4

**d) Quantum Chemical Calculations** ........ 5

**2. Data Analysis: Figures and Tables** ........ 9

**Figure S1**. Absorption spectra of Oxorhodanine, before (*Z* form) and after irradiation at 340 nm CW source to generate the photostationary state containing the *E* isomer. The dashed line shows the wavelength used for selective excitation of *E*. ........ 9

**Figure S2.** a) Transient absorption spectra of the *Z* form of **I** in acetonitrile at different time delays after the pump pulse, with steady-state absorption and emission spectra included on the top for comparison with ground-state bleaching (GSB) and stimulated emission (SE). b) Evolution-associated difference spectra (EADS) derived from global fitting of the corresponding transient absorption data. c) Fits of kinetic traces from Global analysis presented at selected key wavelengths in acetonitrile. ........ 10

**Figure S3.** a) Transient absorption spectra of the *Z* form of **I** in methanol at different time delays after the pump pulse, with steady-state absorption and emission spectra included on the top for comparison with ground-state bleaching (GSB) and stimulated emission (SE). b) Evolution-associated difference spectra (EADS) derived from global fitting of the corresponding transient absorption data. c) Fits of kinetic traces from Global analysis presented at selected key wavelengths in methanol. ........ 11

**Figure S4.** a) Transient absorption spectra of the *Z* form of **I** in THF at different time delays after the pump pulse, with steady-state absorption and emission spectra included on the top for comparison with ground-state bleaching (GSB) and stimulated emission (SE). b) Evolution-

associated difference spectra (EADS) derived from global fitting of the corresponding transient absorption data. c) Fits of kinetic traces from Global analysis presented at selected key wavelengths in THF. ........ 12

**Figure S5.** a) Transient absorption spectra of the *Z* form of **I** in EG at different time delays after the pump pulse, with steady-state absorption and emission spectra included on the top for comparison with ground-state bleaching (GSB) and stimulated emission (SE). Data >700 nm were not collected (sapphire plate continuum) as the focus of the viscosity data was on the excited state decay (SE at 432 nm). b) Evolution-associated difference spectra (EADS) derived from global fitting of the corresponding transient absorption data. Note that SE persists in the second EADS c) Fits of kinetic traces from Global analysis presented at selected key wavelengths in EG. ........ 13

**Figure S6.** a) Transient absorption spectra of the *E* form of **I** in acetonitrile at different time delays after the pump pulse, with steady-state absorption and emission spectra included on the top for comparison with ground-state bleaching (GSB) and stimulated emission (SE). b) Evolution-associated difference spectra (EADS) derived from global fitting of the corresponding transient absorption data. c) Fits of kinetic traces from Global analysis presented at selected key wavelengths in acetonitrile. ........ 14

**Figure S7.** Active space orbitals employed in the XMS-CASPT2/CAS(14,12)/cc-pVDZ single point calculations. For minimum-energy conical intersection optimization (MECI) at the CASSCF(12,11)/cc-pVDZ level, the oxygen lone pair orbital (enclosed by yellow box) was excluded. Isovalue: 0.02 a.u. ........ 15

**Figure S8.** Dominant orbital transitions associated with $S_1$ (ππ*) and $S_2$ (nπ*) states at the $S_0$-min (Z) based on CAS(14,12). ........ 16

**Figure S9.** Key geometrical parameters along important critical points and atom-indexing used. Pyramidalization at $C_{10}$ is required alongside twist to access the $S_1/S_0$-MECI. ........ 17

**Figure S10.** Overlap of the LIIC pathways from FC region to the $S_1/S_0$-MECI for both Z (solid lines) and E isomer (dashed lines) of I. Energies along the LIIC pathway are calculated at XMS-CASPT2/SA5-CAS(14,12)/cc-pVDZ level and reported with respect to the ground state energy at $S_0$-min (Z). The overlap shows the difference between the Pathways for Z and E isomer, with the possibility of a shallow minima near FC region and a small barrier (<0.07 eV) to access the MECI from $S_0$-min (E). ........ 18

**Figure S11.** a) Evolution-associated difference spectra (EADS) derived from the three-component global fitting of the corresponding transient absorption data presented in figure S4a. b) EADS derived from a four-component global-fitting of the same data. c) Fits of kinetic traces from Global analysis with three components, presented at selected key wavelengths in THF. d) Fits of kinetic traces from Global analysis with four components, presented at selected key wavelengths in THF. Note the improved fit specifically in the SE (430 nm) in the first 200 fs. e) Comparison of residuals, retrieved from both the three and four component Global analysis, extracted of the fits of the kinetic trace at 430 nm (stimulated emission). The four-component residuals appear closer to the zero-line around 100 fs, indicating a better fit. ........ 19

**Figure S12.** **(a)** Comparison of the decay of the stimulated emission (selected at peak wavelength) in each solvent studied; Note the similar fast component in each solvent and the slow decay of the SE in EG. (b) The normalised data over the first 2 ps to show the appearance time described in the text for THF. This is absent in ACN. ........ 20

## 1. Experimental section

### a) Steady-State Absorption and Emission

The UV–Visible ground-state absorption spectra were acquired with a PerkinElmer Lambda XLS spectrophotometer with 1 cm path length quartz cuvettes. Emission spectra at ambient temperature were obtained using an Edinburgh Instruments FS5 spectrofluorometer in a right-angle arrangement, with a 2 nm bandwidth for both excitation and emission and maintaining absorbance below 0.1 optical density (OD). The solvent Raman contribution was suntracted.

### b) UV-Visible-NIR fs transient absorption (TA)

The transient absorption apparatus employed has been described in other sources.[1] The pump beams were produced using an 800 nm fundamental output beam from a Spectra Physics-Mai Tai Laser oscillator, amplified by a Ti:Sapphire regenerative amplifier (Spectra Physics-Spitfire ACE). The output pulse, with a duration of 100 fs at 800 nm, a repetition rate of 1 kHz, and an energy of 5 mJ per pulse, was utilized to operate the optical parametric amplifier (OPA, Light Conversion TOPAS Prime), generating a tunable pump pulse for sample excitation. A white light continuum (WLC) probe was produced by focusing a portion of the fundamental 800 nm beam onto a 3 mm thick $CaF_2$ window, resulting in a broadband continuum spanning from 380 to 750 nm. The window was affixed to a translation stage and moved to avert optical damage. In an alternative configuration, another broadband WLC (continuum spanning from 500 to 1300 nm) was produced by pumping the sapphire plate with a 1250 nm NIR probe sourced from another OPA's output. The pump beam's energy at the sample cell was reduced to 300 μW (300 nJ/pulse). All measurements were performed in 1 mm cuvettes with an optical density of less than 1.. Samples were flowed.

### c) Fluorescence Quantum Yield.

The very low fluorescence yield was measured using HBDI (in acetonitrile) as a reference. Both HBDI and oxorhodanine were diluted in acetonitrile to prepare solutions of nearly same OD (~0.09). PerkinElmer Lambda XLS spectrophotometer with 1 cm path length quartz cuvettes was utilized to measure the fluorescence of both. Measured fluorescence were OD corrected before integrating the area under the curve. Afterwards, fluorescence quantum yield was calculated using standard formula,[2] where absolute quantum yield of HBDI was considered to be$10^{-4}$ [3].

### d) Quantum Chemical Calculations

The critical points ($S_0$-min (Z), $S_0$-min (E)) of the oxorhodanine photoswitch on the ground singlet state were optimized at the density functional theory (DFT) level using ωB97x-D functional and 6-31G(d,p) basis set. Frequency calculations were performed at the same level of theory to ensure that the obtained stationary points are the minima. These optimizations were performed using Gaussian16. $S_1/S_0$-minimum-energy conical intersection (MECI) optimization was performed at the complete active space self-consisted field (CASSCF) level using cc-pVDZ basis set and an active space of 12 electrons in 11 orbitals (CAS(12,11)) (shown in Figure S6) with a two-state averaging using BAGEL 1.2.2 package. These three critical point geometries with relevant geometrical parameters are illustrated in Figure. S8. Substantial pyramidalization of $C_{10}$ is required to reach the $S_1/S_0$-MECI in addition to the twist motion, making it part of a twisted-pyramidalized conical intersection seam space. Linear interpolations were carried out in internal coordinates (LIIC) between the $S_0$-min (Z) and the $S_1/S_0$-MECI, and between the $S_1/S_0$-MECI and the $S_0$-min (E). Vertical excitation energies (VEE) and oscillator strengths were calculated along the LIIC at the high-level extended multistate complete active space second-order perturbation (XMS-CASPT2) theory the using OpenMolcas 25.10. Five singlet states and three triplet states (SA(5/3)) were averaged using an active space of 14 electrons in 12 orbitals (Figure S6) and cc-pVDZ basis set. IPEA shift was set to 0 a.u., whilst the imaginary shift was set to 0.3 a.u. to remove intruder states. Spin-orbit coupling matrix elements (SOCME) were also calculated using the XMS-CASPT2 wavefunctions along the LIIC. The maximum of the SOCME elements between a singlet and a triplet state was used as the SOC value between a singlet and a triplet state.

e) Synthesis and Characterization

### Experimental

Thiazolidine-2,4-dione (1 g, 8.5 mmol), 4-methyl-1H-imidazole-5-carboxaldehyde (1g, 9 mmol) and urea (55 mg, 0.9 mmol) were refluxed in methanol (30 ml) for 2 hrs. A yellow solution was obtained. After cooling, the solution was filtered through celite and the solvent left to evaporate slowly. A small amount of orange crystals (200 mg, 11%) was isolated.

$^{1}H$ NMR (400 MHz, DMSO-$d_6$) δ 12.50 (s, 1H), 12.05 (s, 1H), 7.78 (s, 1H), 7.59 (s, 1H), 2.36 (s, 3H) ppm.

$^{13}C$ NMR (101 MHz, DMSO) δ 171.38, 167.64, 136.55, 133.33, 131.83, 122.84, 117.60, 9.16 ppm.

MALDI-TOF m/z 209.0635 [M], calculated $C_8H_7O_2SN_3$ m/z 209.0245

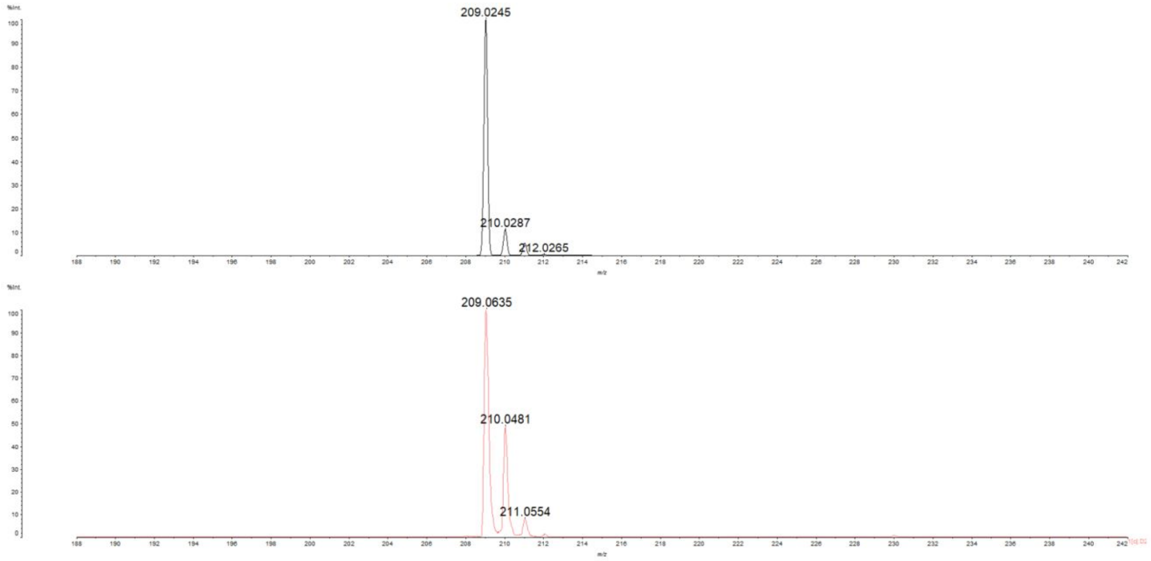


MALDI-TOF. Compound **1**. Top trace: theoretical [M]. Bottom: experimental

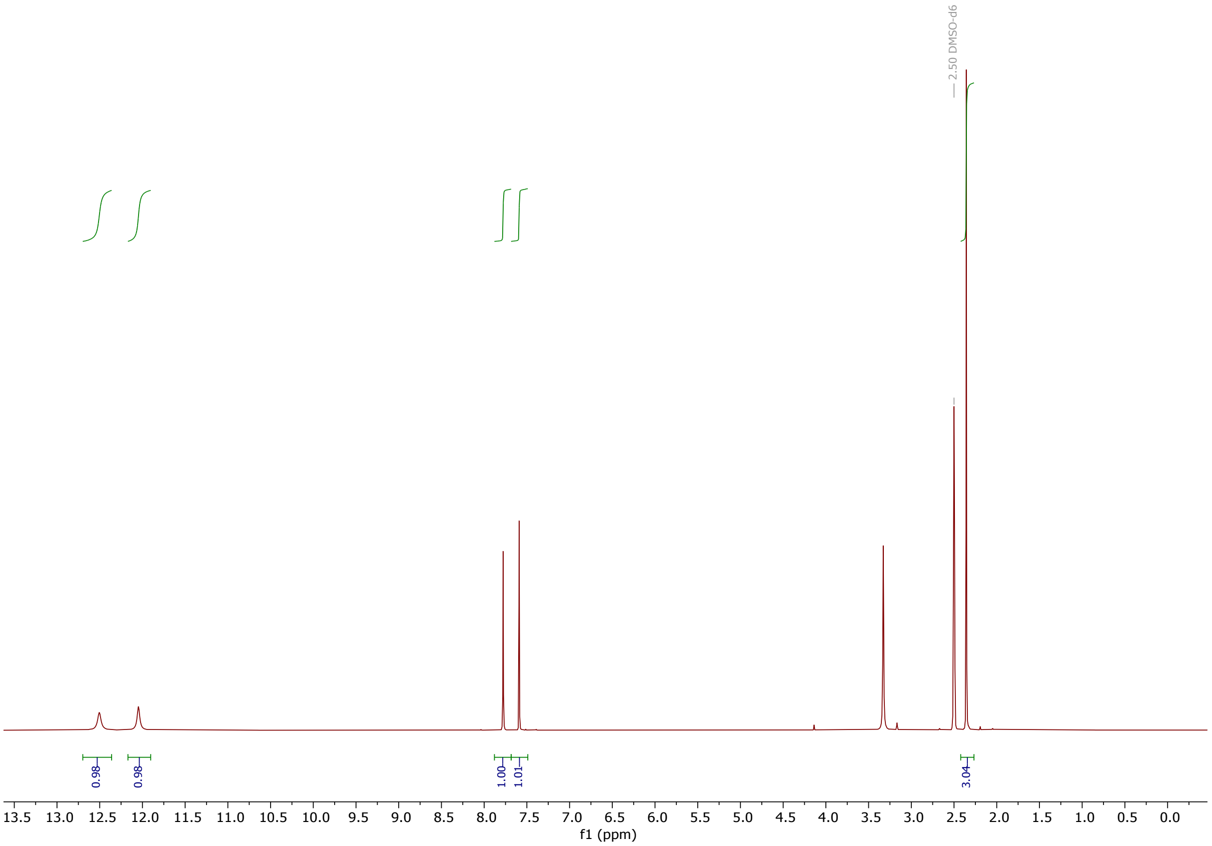


Compound **1**. $^{1}H$ NMR (400 MHz, DMSO-$d_6$) spectrum

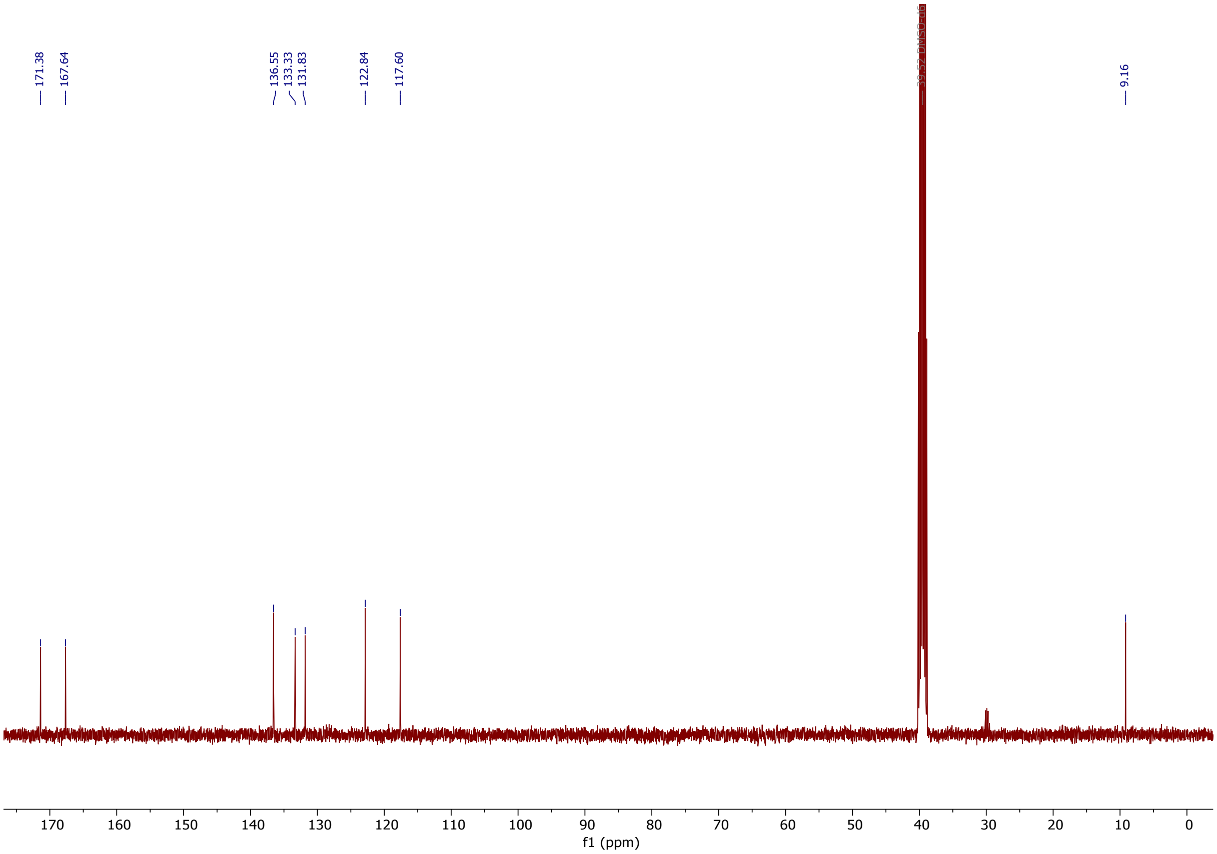


Compound **1**. $^{13}C$ NMR (101 MHz, DMSO) spectrum

## 2. Data Analysis: Figures and Tables

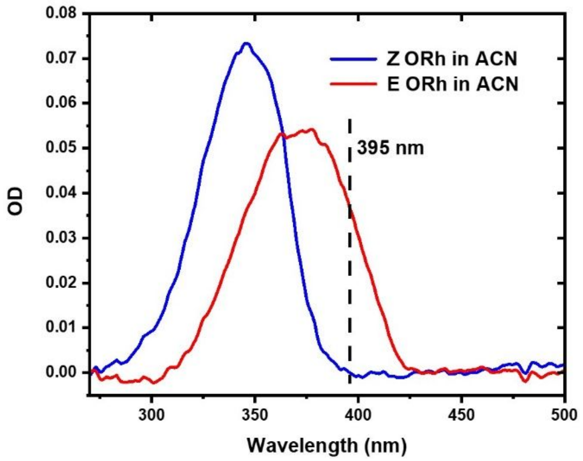


**Figure S1**. Absorption spectra of Oxorhodanine, before (*Z* form) and after irradiation at 340 nm CW source to generate the photostationary state containing the *E* isomer. The dashed line shows the wavelength used for selective excitation of *E*.

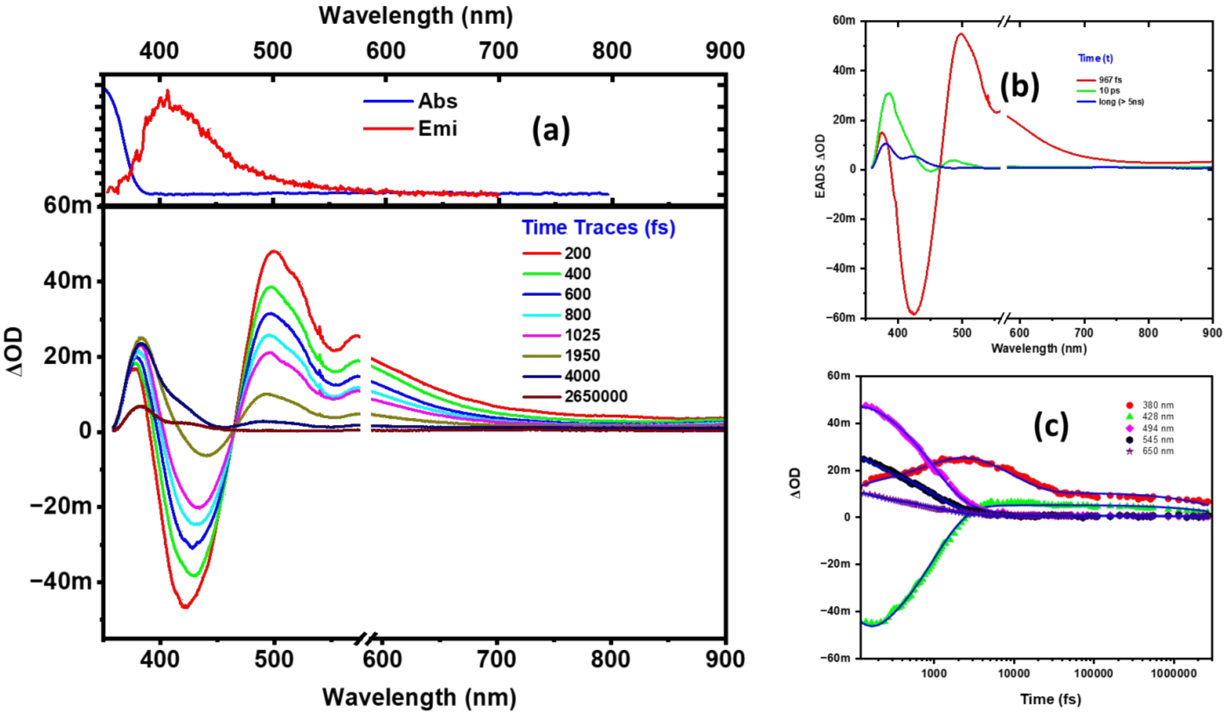


**Figure S2.** a) Transient absorption spectra of the *Z* form of **I** in acetonitrile at different time delays after the pump pulse, with steady-state absorption and emission spectra included on the top for comparison with ground-state bleaching (GSB) and stimulated emission (SE). b) Evolution-associated difference spectra (EADS) derived from global fitting of the corresponding transient absorption data. c) Fits of kinetic traces from Global analysis presented at selected key wavelengths in acetonitrile.

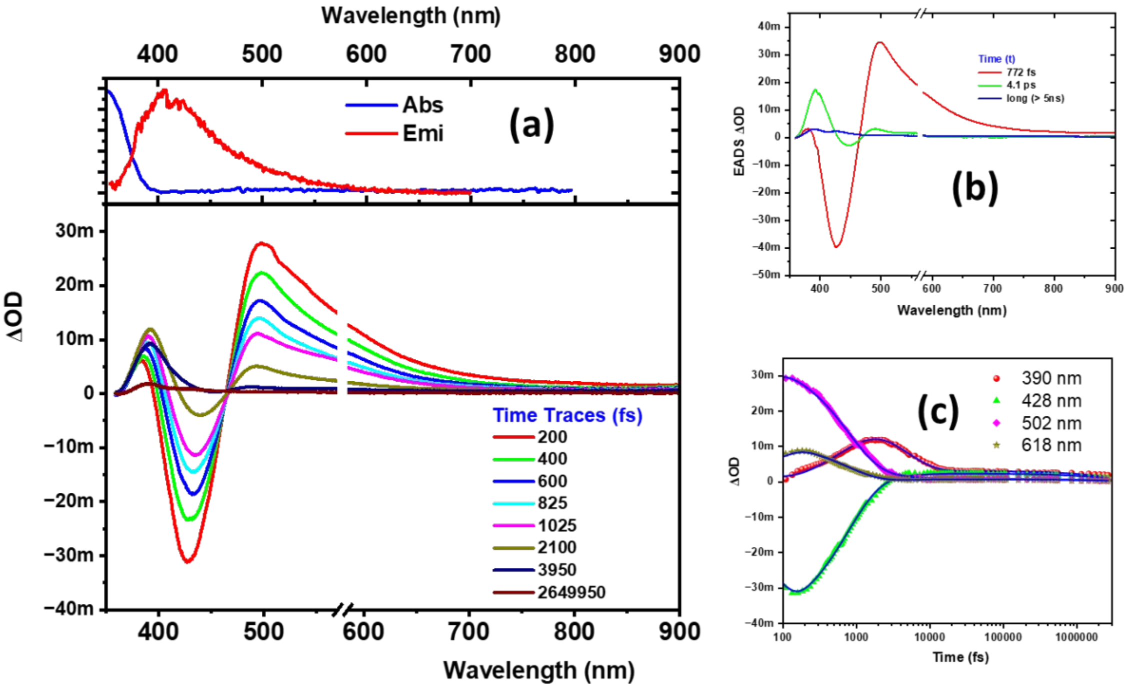


**Figure S3.** a) Transient absorption spectra of the *Z* form of **I** in methanol at different time delays after the pump pulse, with steady-state absorption and emission spectra included on the top for comparison with ground-state bleaching (GSB) and stimulated emission (SE). b) Evolution-associated difference spectra (EADS) derived from global fitting of the corresponding transient absorption data. c) Fits of kinetic traces from Global analysis presented at selected key wavelengths in methanol.

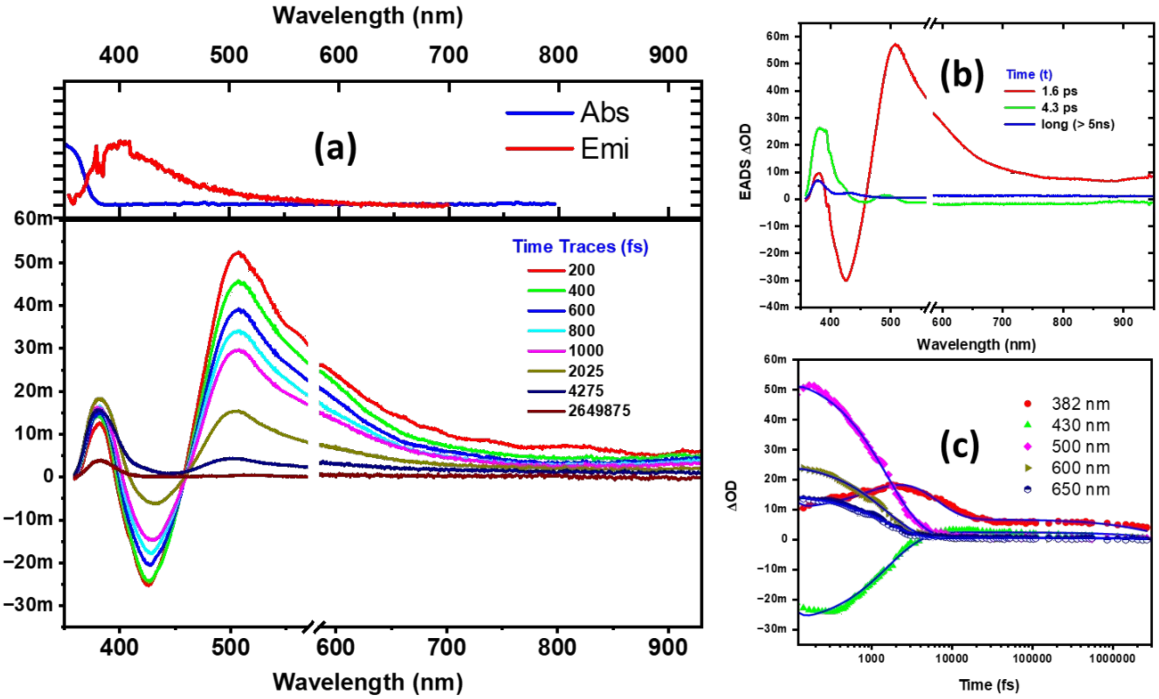


**Figure S4.** a) Transient absorption spectra of the *Z* form of **I** in THF at different time delays after the pump pulse, with steady-state absorption and emission spectra included on the top for comparison with ground-state bleaching (GSB) and stimulated emission (SE). b) Evolution-associated difference spectra (EADS) derived from global fitting of the corresponding transient absorption data. c) Fits of kinetic traces from Global analysis presented at selected key wavelengths in THF.

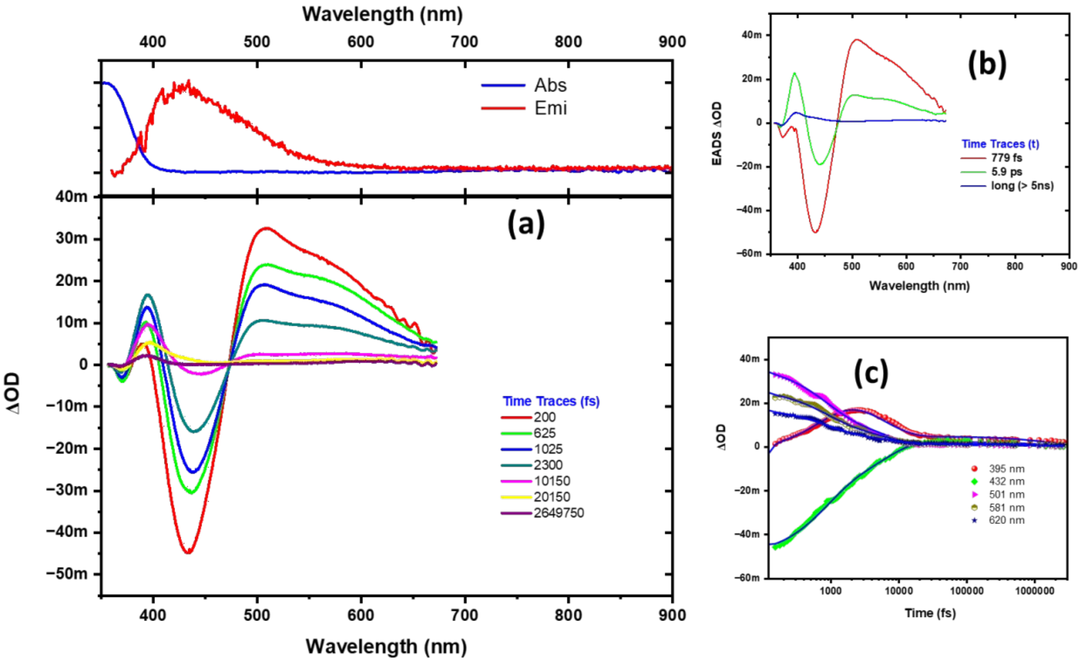


**Figure S5.** a) Transient absorption spectra of the *Z* form of **I** in EG at different time delays after the pump pulse, with steady-state absorption and emission spectra included on the top for comparison with ground-state bleaching (GSB) and stimulated emission (SE). Data >700 nm were not collected (sapphire plate continuum) as the focus of the viscosity data was on the excited state decay (SE at 432 nm). b) Evolution-associated difference spectra (EADS) derived from global fitting of the corresponding transient absorption data. Note that SE persists in the second EADS c) Fits of kinetic traces from Global analysis presented at selected key wavelengths in EG.

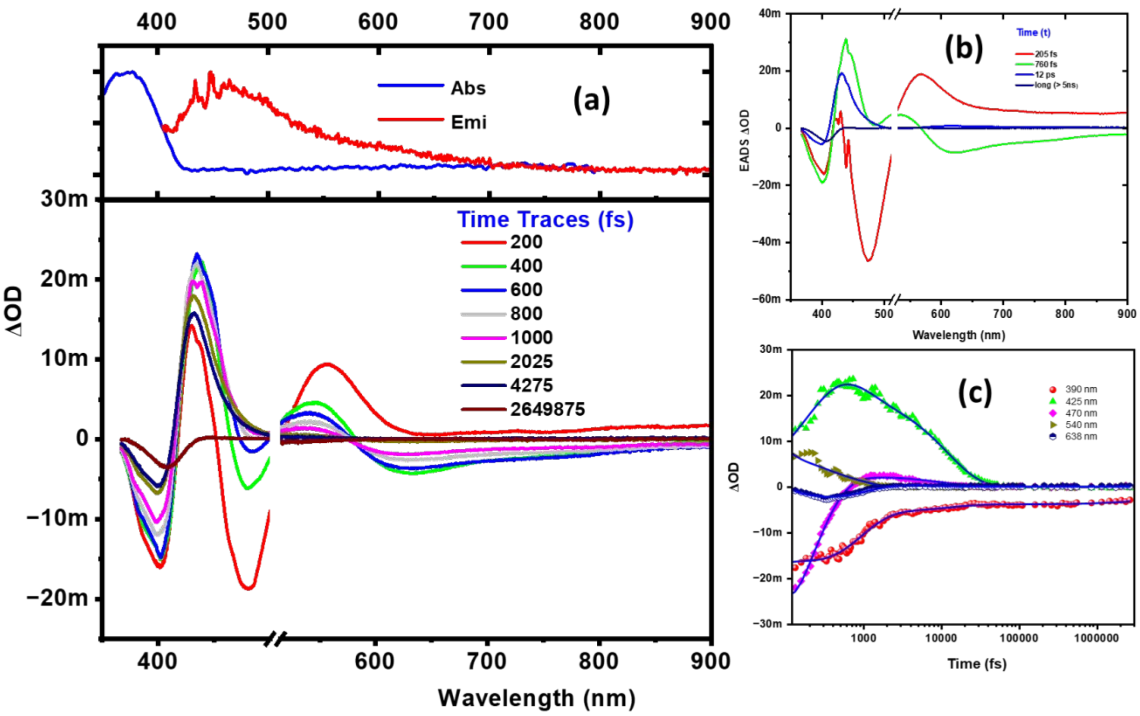


**Figure S6.** a) Transient absorption spectra of the *E* form of **I** in acetonitrile at different time delays after the pump pulse, with steady-state absorption and emission spectra included on the top for comparison with ground-state bleaching (GSB) and stimulated emission (SE). b) Evolution-associated difference spectra (EADS) derived from global fitting of the corresponding transient absorption data. c) Fits of kinetic traces from Global analysis presented at selected key wavelengths in acetonitrile.

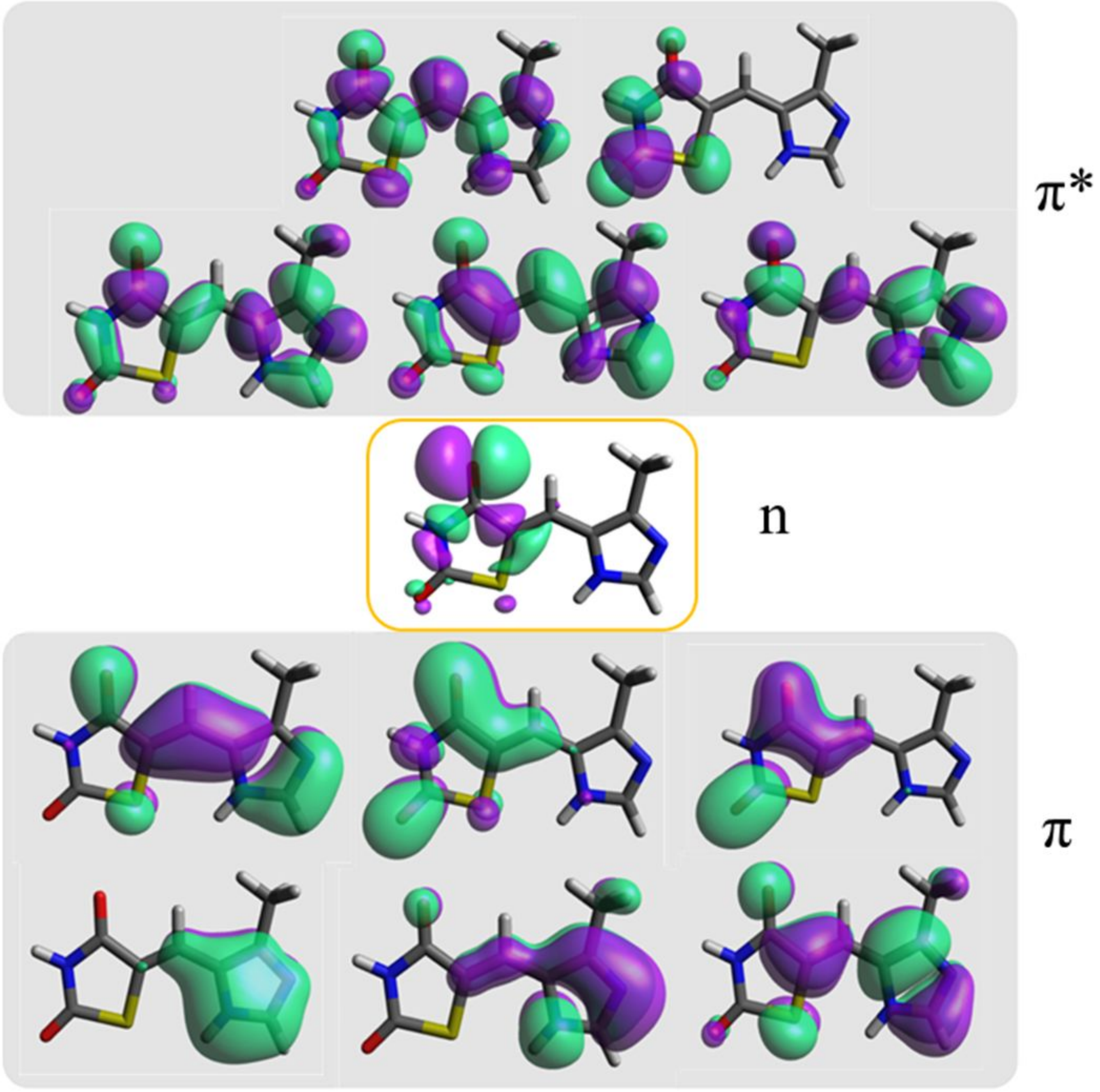


**Figure S7.** Active space orbitals employed in the XMS-CASPT2/CAS(14,12)/cc-pVDZ single point calculations. For minimum-energy conical intersection optimization (MECI) at the CASSCF(12,11)/cc-pVDZ level, the oxygen lone pair orbital (enclosed by yellow box) was excluded. Isovalue: 0.02 a.u.

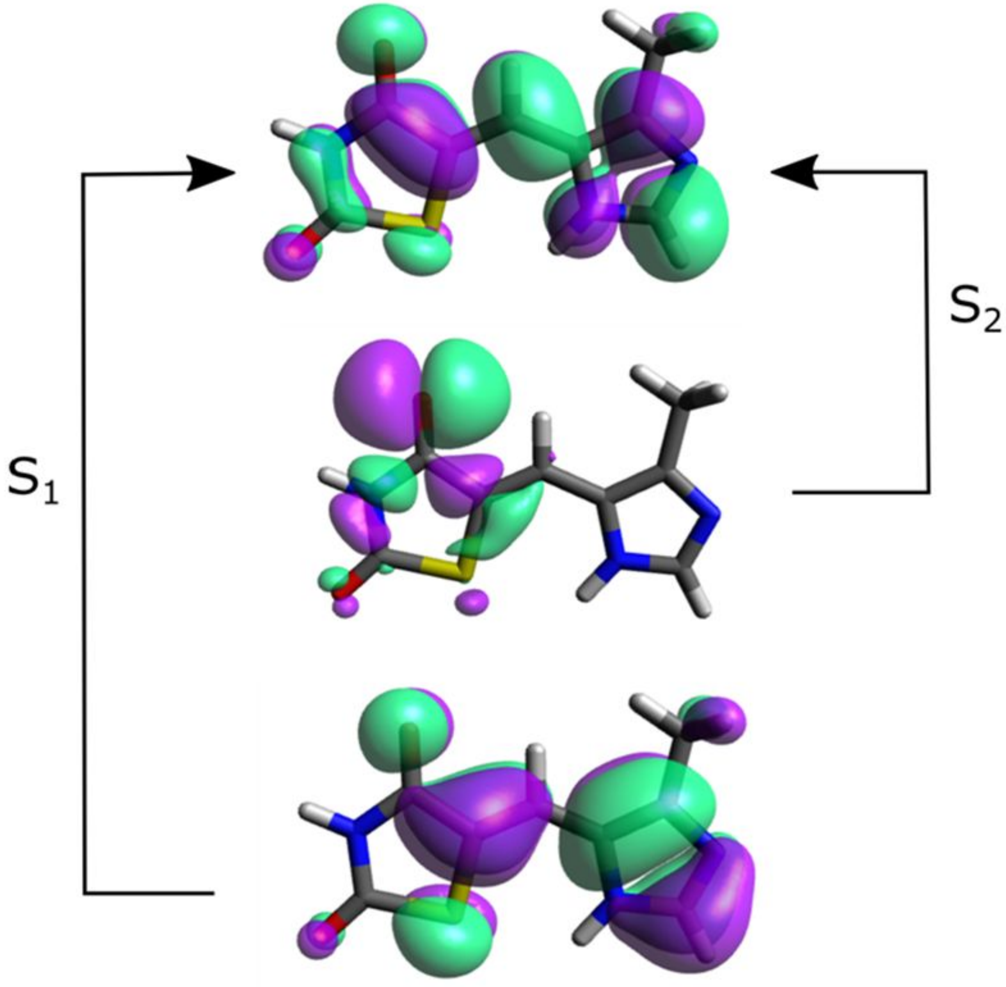


**Figure S8.** Dominant orbital transitions associated with $S_1$ ($\pi\pi^*$) and $S_2$ ($n\pi^*$) states at the $S_0$-min (Z) based on CAS(14,12).

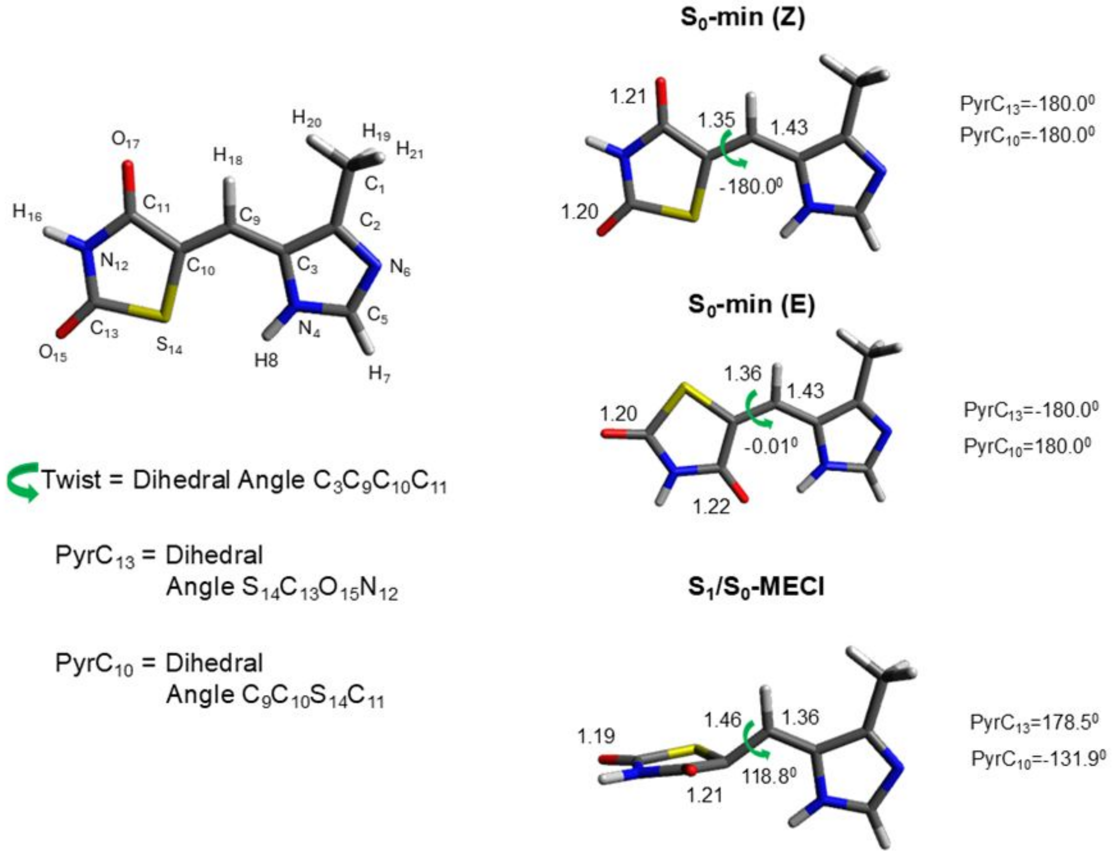


**Figure S9.** Key geometrical parameters along important critical points and atom-indexing used. Pyramidalization at $C_{10}$ is required alongside twist to access the $S_1/S_0$-MECI.

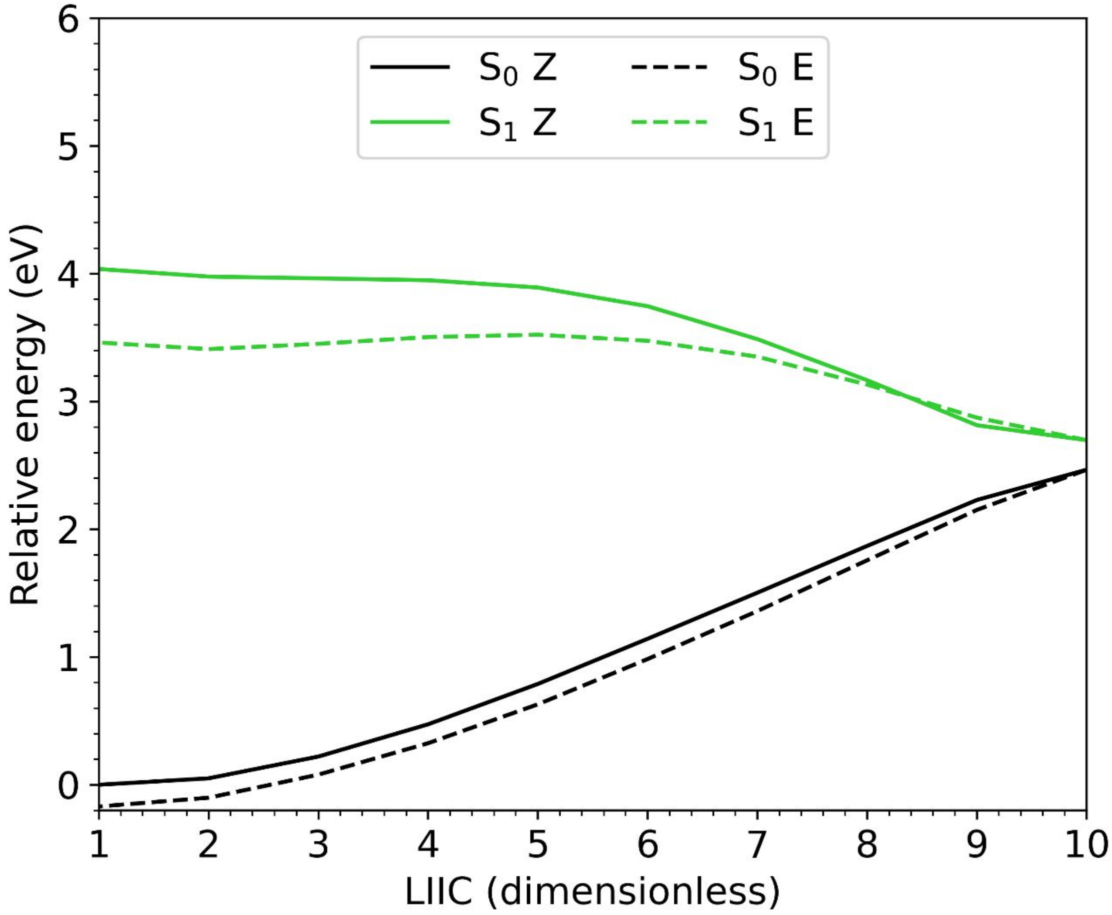


**Figure S10.** Overlap of the LIIC pathways from FC region to the $S_1/S_0$-MECI for both Z (solid lines) and E isomer (dashed lines) of I. Energies along the LIIC pathway are calculated at XMS-CASPT2/SA5-CAS(14,12)/cc-pVDZ level and reported with respect to the ground state energy at $S_0$-min (Z). The overlap shows the difference between the Pathways for Z and E isomer, with the possibility of a shallow minima near FC region and a small barrier (<0.07 eV) to access the MECI from $S_0$-min (E).

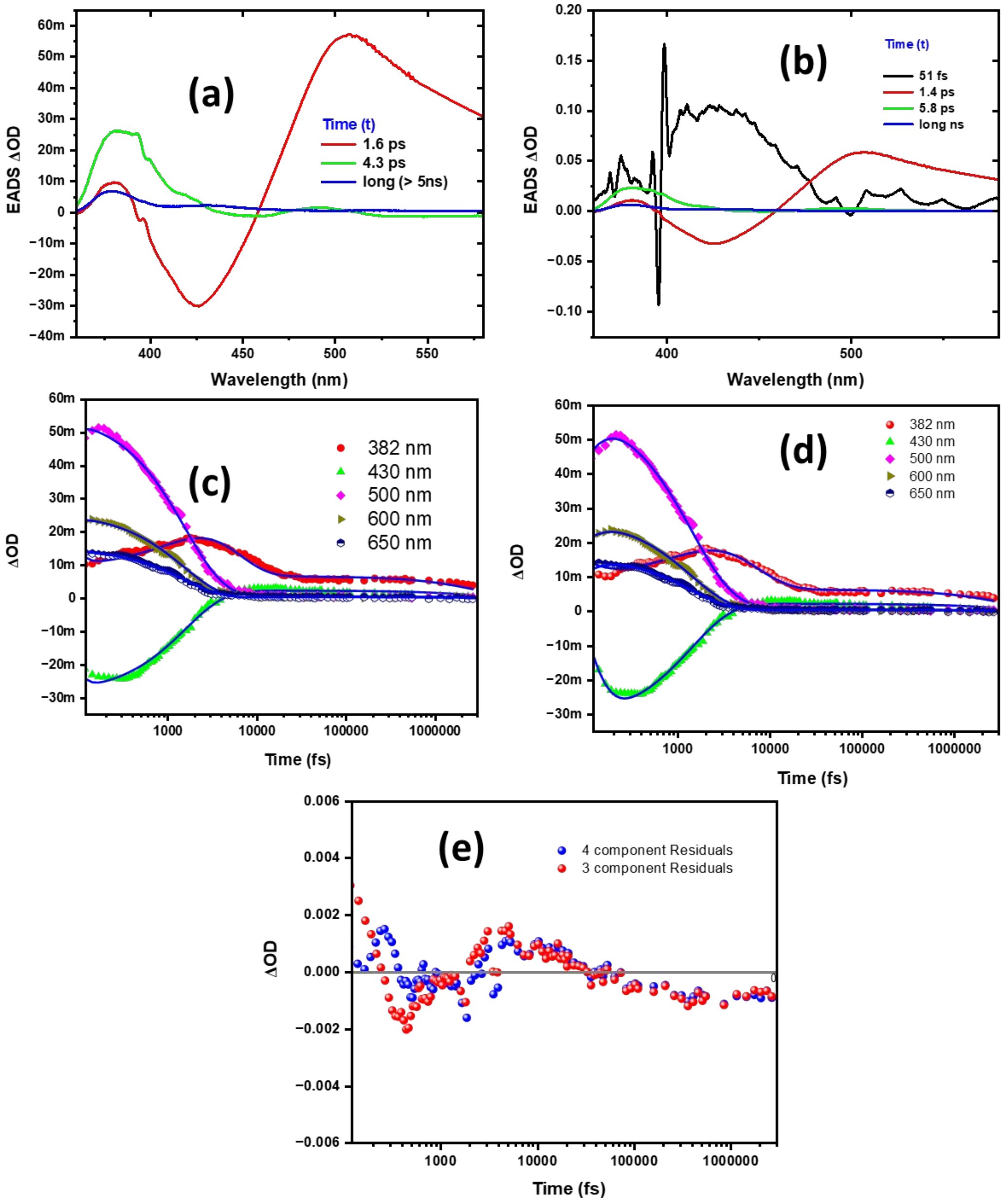


**Figure S11.** a) Evolution-associated difference spectra (EADS) derived from the three-component global fitting of the corresponding transient absorption data presented in figure S4a. b) EADS derived from a four-component global-fitting of the same data. c) Fits of kinetic traces from Global analysis with three components, presented at selected key wavelengths in THF. d) Fits of kinetic traces from Global analysis with four components, presented at selected key wavelengths in THF. Note the improved fit specifically in the SE (430 nm) in the first 200 fs. e) Comparison of residuals, retrieved from both the three and four component Global analysis, extracted of the fits of the kinetic trace at 430 nm (stimulated emission). The four-component residuals appear closer to the zero-line around 100 fs, indicating a better fit.

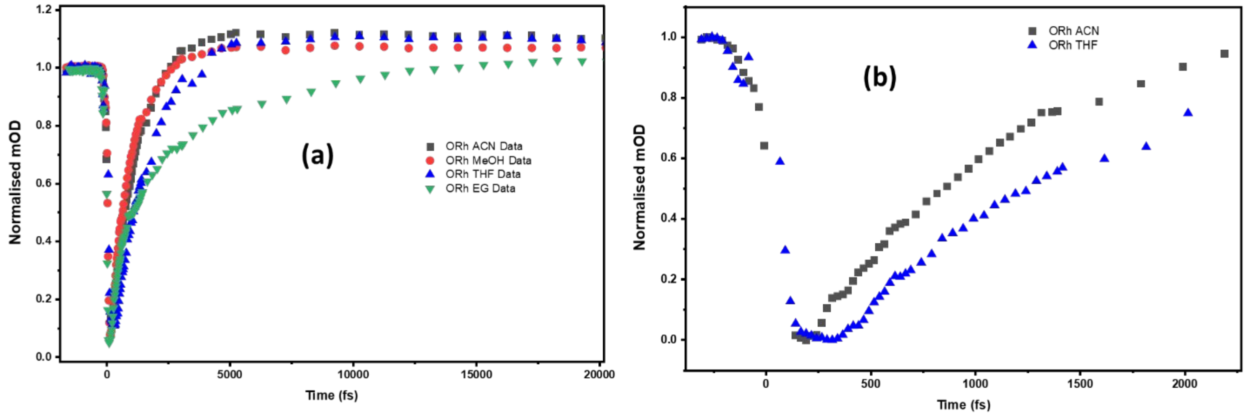


**Figure S12.** **(a)** Comparison of the decay of the stimulated emission (selected at peak wavelength) in each solvent studied; Note the similar fast component in each solvent and the slow decay of the SE in EG. (b) The normalised data over the first 2 ps to show the appearance time described in the text for THF. This is absent in ACN.

**Optimized critical point coordinates (in Å) at DFT/ωB97X-D/6-31G(d,p) level:**

**$S_0$-min (Z):**

| | | | |
|---|---|---|---|
| C | 3.227617 | 2.389500 | -0.034385 |
| C | 1.743481 | 2.542935 | -0.034097 |
| C | 0.767495 | 1.554267 | -0.018243 |
| N | -0.432723 | 2.250245 | -0.026406 |
| C | -0.130411 | 3.566098 | -0.045766 |
| N | 1.167468 | 3.781148 | -0.051192 |
| H | -0.892879 | 4.332088 | -0.055618 |
| H | -1.360937 | 1.859194 | -0.018982 |
| C | 0.922892 | 0.133431 | 0.001943 |
| C | -0.002024 | -0.844803 | 0.017429 |
| C | 0.414444 | -2.266436 | 0.037281 |
| N | -0.717871 | -3.078421 | 0.051264 |
| C | -1.967831 | -2.497779 | 0.044789 |
| S | -1.765765 | -0.705296 | 0.018113 |
| O | -3.024854 | -3.064529 | 0.054886 |
| H | -0.633274 | -4.084882 | 0.065712 |
| O | 1.548200 | -2.691391 | 0.041794 |
| H | 1.948657 | -0.227698 | 0.005531 |
| H | 3.657699 | 2.887414 | 0.838898 |
| H | 3.534837 | 1.342004 | -0.019220 |
| H | 3.654605 | 2.860948 | -0.923730 |

**$S_0$-min (E):**

| | | | |
|---|---|---|---|
| C | -0.066685 | 0.023647 | -0.000747 |
| C | 0.257144 | -1.433425 | 0.019961 |
| C | -0.608788 | -2.529293 | 0.037669 |
| N | 0.219822 | -3.634052 | 0.052226 |
| C | 1.481641 | -3.180225 | 0.043187 |

| | | | |
|---|---|---|---|
| N | 1.548189 | -1.858171 | 0.023679 |
| H | 2.335100 | -3.843194 | 0.051212 |
| H | -0.142767 | -4.587703 | 0.066646 |
| C | -2.033760 | -2.533098 | 0.040306 |
| C | -2.940980 | -3.542335 | 0.056297 |
| C | -2.698605 | -4.993760 | 0.076364 |
| N | -3.905056 | -5.668039 | 0.087067 |
| C | -5.090534 | -4.948995 | 0.078494 |
| S | -4.692245 | -3.208185 | 0.054464 |
| O | -6.195884 | -5.418169 | 0.086414 |
| H | -3.929056 | -6.678151 | 0.100979 |
| O | -1.629836 | -5.592029 | 0.083164 |
| H | -2.452890 | -1.529424 | 0.026681 |
| H | 0.364946 | 0.491780 | -0.889564 |
| H | -1.142112 | 0.213790 | -0.001888 |
| H | 0.367644 | 0.517469 | 0.872726 |

**$S_1/S_0$-MECI coordinate (in Å) at SA2-CASSCF(12,11)/cc-pVDZ level:**

| | | | |
|---|---|---|---|
| C | 4.253324575202e-02 | 4.653440870971e-02 | 1.140929163852e-02 |
| C | -5.732461698459e-01 | -6.191482377814e-01 | -1.176361090076e+00 |
| C | -1.541948885513e+00 | -1.694973187270e+00 | -1.177887113941e+00 |
| N | -1.764192977827e+00 | -1.923069528376e+00 | -2.528294247570e+00 |
| C | -1.022430449477e+00 | -1.093989647898e+00 | -3.212222272900e+00 |
| N | -2.827941284365e-01 | -2.758816508659e-01 | -2.412522178301e+00 |
| H | -9.971547123826e-01 | -1.063909251291e+00 | -4.288181148243e+00 |
| H | -2.393122401969e+00 | -2.655049239790e+00 | -2.822933424225e+00 |
| C | -2.176499434959e+00 | -2.440039708733e+00 | -2.281269835639e-01 |
| C | -3.130807929652e+00 | -3.477004590723e+00 | -6.259017952929e-01 |
| C | -2.788476727412e+00 | -4.819120014456e+00 | -2.253413206730e-01 |
| N | -3.938655091558e+00 | -5.559326184772e+00 | 6.478747049071e-02 |
| C | -5.124009895768e+00 | -4.892194383307e+00 | 2.155451830429e-01 |
| S | -4.863466131863e+00 | -3.170381938020e+00 | -1.943464523222e-01 |
| O | -6.166247287174e+00 | -5.370608860592e+00 | 5.405584241379e-01 |
| H | -3.871396666888e+00 | -6.519589900127e+00 | 3.341161457984e-01 |
| O | -1.681554063517e+00 | -5.300551148299e+00 | -1.282586163538e-01 |
| H | -1.925470243131e+00 | -2.228181241681e+00 | 8.122093926229e-01 |
| H | -1.784064099289e-01 | 1.113297713695e+00 | -1.032899537644e-02 |
| H | -3.280899763287e-01 | -3.725553489991e-01 | 9.435785031936e-01 |

| | |
|---|---|
| H | 1.124993191951e+00 -7.309993550365e-02 -2.379040063654e-02 |

## References

[1] Fatima, A.; Chakraborty, P.; Xu, X.; Jones, G. A.; Chambrier, I.; Logan, G.; Cammidge, A. N.; Smith, T.; Hall, C. R.; Meech, S. R. Complex Multistate Photophysics of a Rhodanine Photoswitch. *Angew. Chem. Int. Ed.* **2025**, *64*, e202506137

[2] S. R. Meech and D. Phillips, J. Photochem., **1983**, *23*, 193-217

[3] S. A. Boulanger, C. Chen, L. Tang, L. Zhu, N. S. Baleeva, I. N. Myasnyanko, M. S. Baranov and C. Fang *Phys. Chem. Chem. Phys.*, 2021,**23**, 14636-14648
